# Sydney Phylogenetics Workshop: Lessons From the Past 15 Years

**DOI:** 10.1002/ece3.74242

**Published:** 2026-09-07

**Authors:** Simon Y. W. Ho

**Affiliations:** ^1^ School of Life and Environmental Sciences University of Sydney Sydney New South Wales Australia

**Keywords:** Bayesian phylogenetics, molecular dating, molecular evolution, phylogenetic analysis, workshop

## Abstract

Phylogenetic analysis forms an important component of research in the life sciences, enabling the study of evolutionary relationships, timescales, patterns, and processes. Phylogenetic trees are used in a wide variety of research fields, including molecular ecology, taxonomy and systematics, conservation genetics, and microbiology. Accordingly, there is a strong demand for accessible training in phylogenetic analysis, especially among postgraduate students and other early career researchers. Although there are several regular workshops that provide opportunities for training in phylogenetics, these are predominantly held in Europe and North America, posing logistical and financial barriers for prospective participants in the Southern Hemisphere and Asia‐Pacific region. The Sydney Phylogenetics Workshop has aimed to address this gap by offering an annual training opportunity suitable for early career researchers. The workshop blends theory with practical exercises using industry‐standard software, while its content has evolved over its 15‐year history to meet the changing needs of attendees. In this article, we provide an outline of the content and format of the workshop, the challenges associated with running such an event on a minimal budget, and the measures taken to improve accessibility and reduce environmental impact. We hope that the workshop provides a useful model for organizing effective training opportunities for early career researchers in the life sciences.

## Graphical abstract

Phylogenetic analysis forms an important component of research in the life sciences. The Sydney Phylogenetics Workshop has offered an annual training opportunity suitable for early career researchers. In this article, we provide an outline of the content and format of the workshop, the challenges associated with running such an event on a minimal budget, and the measures taken to improve accessibility and reduce environmental impact.

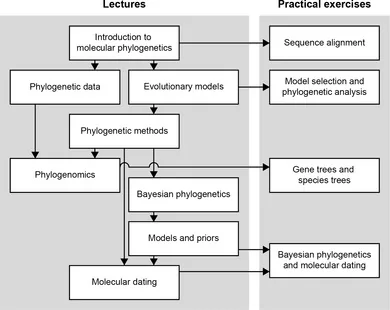

## 1 Introduction

Phylogenetic analysis, or the process of inferring the evolutionary tree of organisms of interest, is an important element of research in evolutionary biology and other life sciences (Yang and Rannala 2012). Most phylogenetic analyses are now based on DNA sequence data, although morphological and phenotypic traits, protein sequences, and other biological data can also be used for this purpose. Phylogenetic trees provide the basis for studies of evolutionary relationships, rates of genomic and phenotypic change, evolutionary timescales, macroevolutionary processes, biogeographic history, and infectious disease dynamics (Wiley 2010). Accordingly, phylogenetic inference methods represent important analytical tools for a broad cross‐section of the life and health sciences, including molecular ecology, conservation genetics, evolutionary biology, microbiology, and pathogen biology.

Given the central importance of evolutionary trees, there is a widespread need for phylogenetic literacy as well as specialized skills in phylogenetic analysis (Baum et al. 2005; Baum and Offner 2008; Gregory 2008). The relevant concepts and skills are typically introduced in undergraduate biology courses, but confidence and competency are rarely achieved at this stage (Sandvik 2008; Young et al. 2013). Phylogenetic analysis is often viewed as a technically challenging exercise, and such skills are not usually gained until the postgraduate or postdoctoral level. Accordingly, there is perennial demand for training opportunities in phylogenetic analysis, particularly among early career researchers.

Some training workshops on phylogenetics and evolutionary analysis are offered on a regular basis, such as Taming the BEAST (Barido‐Sottani et al. 2018), the Workshop on Molecular Evolution at Woods Hole, the Bodega Bay Applied Phylogenetics Workshop, and the Evomics Workshop (Barth et al. 2023). However, these tend to be held in Europe and North America, where the majority of instructors and attendees are based. Given the financial costs, time commitments, and carbon emissions involved in attending these workshops, they are often inaccessible or unappealing to early career researchers based in the Southern Hemisphere or in the Asia‐Pacific region. Even if workshops are held online, the virtual nature of these events and differences in time zones can be prohibitive to meaningful participation, engagement with learning materials, and interaction with instructors and other attendees (Meyer et al. 2021; Raby and Madden 2021b; Barido‐Sottani et al. 2022).

The Sydney Phylogenetics Workshop has aimed to meet some of these training needs by providing an introduction to phylogenetic analysis that is aimed at early career researchers. Held annually in Sydney, Australia, the workshop presents content that comprises a mixture of theory and practice, with a consistent emphasis on pragmatism. By including practical exercises using a range of software, the workshop encourages participants to take the first steps towards adopting some of the most widely used methods, models, and programs for their own research.

Here, we provide an outline of the Sydney Phylogenetics Workshop and some of the lessons learned over its 15‐year history. This overview will focus on the evolution of the workshop syllabus over the course of its existence, the composition of recent attendees, and the measures that have been taken to improve accessibility. This article is intended to present an illustration of a long‐running and well‐attended workshop that has been delivered on a very limited budget, which we hope will provide information, inspiration, and motivation for those wishing to organize similar courses.

## 2 Evolution of the Workshop Syllabus

The Sydney Phylogenetic Workshop was initiated in 2010 as a specialized training course in Bayesian phylogenetic inference, building on earlier workshops that had been held in Canberra, Australia. At the time, there was widespread use of maximum parsimony, distance‐based methods, and maximum likelihood in phylogenetic studies. However, the Bayesian approach was seeing rapid growth in evolutionary biology, having been introduced to phylogenetics in the late 1990s (Rannala and Yang 1996; Mau and Newton 1997).

Bayesian phylogenetic methods went through substantial development and expansion in the late 2000s, with new models presenting valuable opportunities for reconstructing evolutionary processes. These models included relaxed molecular clocks that could account for evolutionary rate variation across lineages (Drummond et al. 2006; Rannala and Yang 2007), calibration priors that could be used to incorporate fossil age information into evolutionary trees for the purpose of molecular dating (Yang and Rannala 2006; Ho and Phillips 2009), Bayesian skyline plots for inferring demographic history (Drummond et al. 2005), and phylogeographic models for reconstructing ancestral location states (Lemey et al. 2009). Such additions enabled researchers to carry out a range of evolutionary analyses in an integrated statistical framework, with joint estimation of model parameters and appropriate quantification of uncertainty (Nascimento et al. 2017).

Over time, it became clear that there was widespread demand for a workshop that could provide a broader introduction to phylogenetic analysis, particularly for early career researchers. In response, the focus of the Sydney Phylogenetics Workshop shifted to a more introductory level to include content on data collection and preparation, “tree thinking”, and the principles of phylogenetic inference. The workshop content was also expanded to include other major phylogenetic approaches such as maximum parsimony, distance‐based methods, and maximum likelihood. These additions called for practical exercises using a wider range of software, particularly programs with graphical user interfaces such as MEGA (Kumar et al. 2024).

More recently, phylogenetic practice has shifted towards the use of much larger, genome‐scale data sets. These phylogenomic data sets present considerable challenges in terms of computational expense and analytical complexity (Young and Gillung 2020; Liu et al. 2015). As a consequence, there has emerged a strong demand for skills and training in phylogenomic and population genomic analysis. In 2017, the Sydney Phylogenetics Workshop began to incorporate new material about different genomic data types, approaches to data selection and curation, and species‐tree methods for analyzing phylogenomic data sets.

## 3 Current Workshop Organization and Syllabus

**FIGURE 1:**
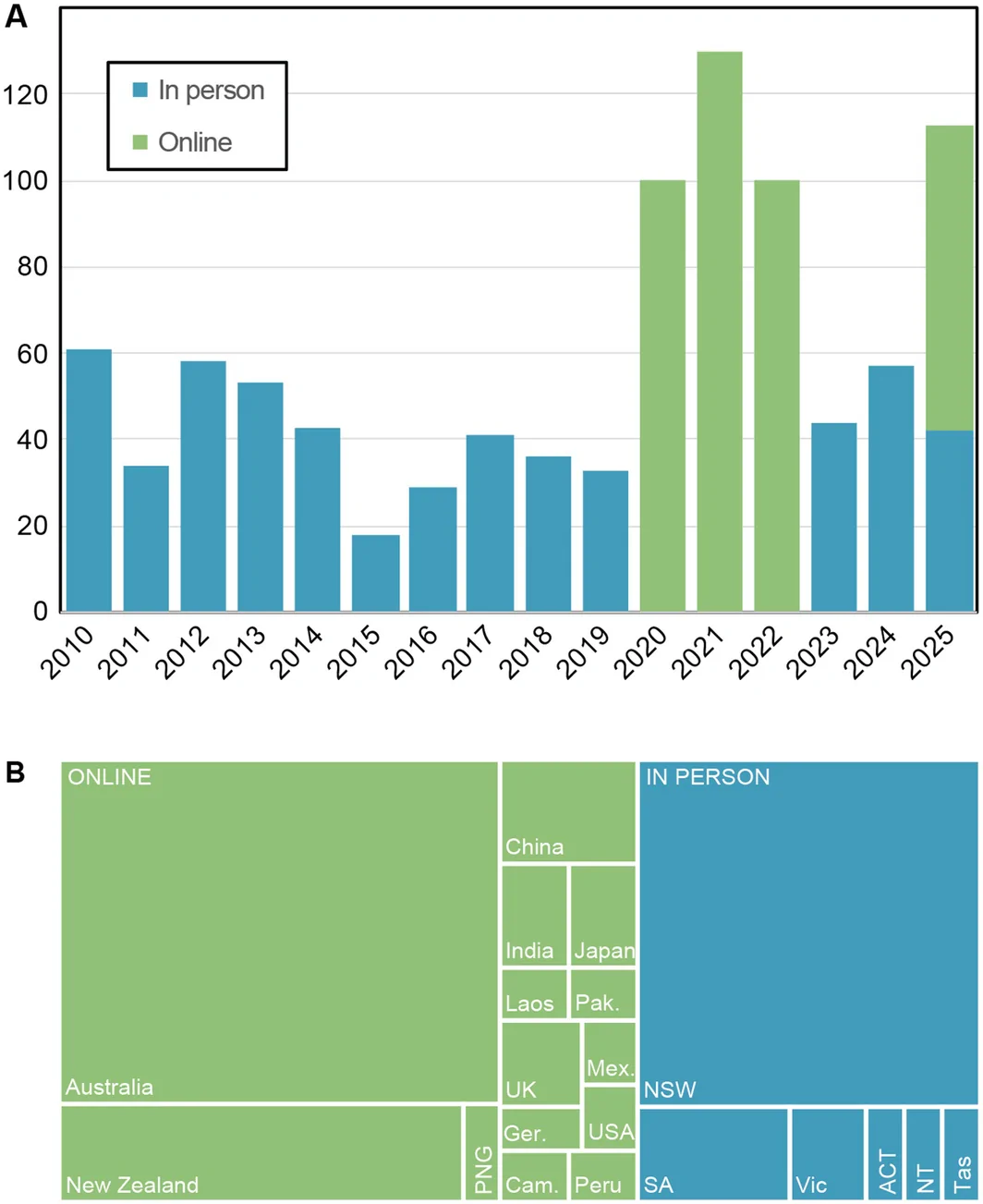
(A) Numbers and proportions of in‐person and online attendees at the Sydney Phylogenetics Workshop from 2010 to 2025. (B) Treemap showing location of in‐person and online attendees at the 2025 workshop. All in‐person attendees were based at institutions within Australia. Country abbreviations: Cam. = Cameroon, Ger. = Germany, Mex. = Mexico, Pak. = Pakistan, UK = United Kingdom, USA = United States of America. Australian state and territory abbreviations are: ACT = Australian Capital Territory, NSW = New South Wales, NT = Northern Territory, SA = South Australia, Tas = Tasmania, Vic = Victoria.

The workshop has been advertised annually through a number of channels, including local institutions, professional societies in Australasia, and social media. Advertisement begins locally and expands to capture larger national and international networks until the number of workshop applicants reaches a chosen level. Prospective attendees are asked to complete a short online application form that captures basic information about institutional affiliation, location, field of research, and level of experience in phylogenetics. These data allow the instructors to offer places to participants who would derive the most benefit from the workshop, and to tailor the workshop material according to the interests of attendees. In most years, the workshop has been able to accommodate the majority of applicants even though places are typically capped at 40 or 50 in‐person attendees. When the workshop has been run in an online or hybrid format, there has usually been a cap of 100 online attendees (Figure 1). Although live‐streamed lectures can potentially accommodate much larger numbers of viewers, this cap is implemented to ensure that appropriate assistance and timely responses can be provided to online workshop attendees.

Attendance at the Sydney Phylogenetics Workshop has invariably been free for all registrants, including both in‐person and online attendees. The costs of running the workshop are minimal because organizers and instructors are based locally and have contributed their time and expertise on a voluntary basis. Time commitments from volunteer instructors are small and usually involve delivering a single talk or assisting with one or two practical exercises. The event is hosted at a university venue, which does not incur any substantial cost. Catering represents the largest expenditure item and is usually covered by internal funding or by small grants from societies or similar sources.

Although the workshop is usually hosted in a computer lab or similar teaching facility, participants are requested to use their own laptop computers so that there is no need to obtain permissions to access university resources. Prior to attending the workshop, participants are asked to install the relevant computer programs used for the practical exercises, all of which are freely available online. This allows attendees to complete the practical exercises in a familiar computing environment, reduces the impact of installation problems and related delays, and enables the scheduled time to be used more efficiently. Attendees can then readily use the installed software to analyze their own data after the workshop.

**FIGURE 2:**
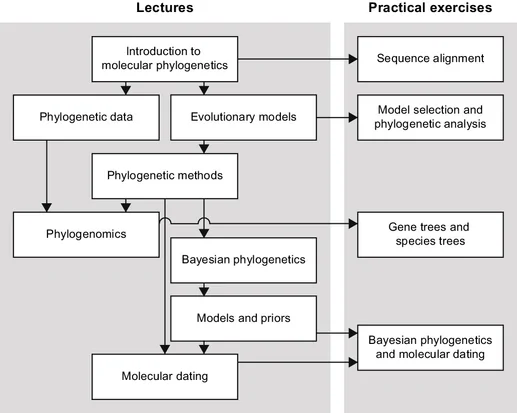
Relationships among the lectures and practical exercises of the 16th annual Sydney Phylogenetics Workshop, held in June 2025. The workshop components were delivered across 14 contact hours.

The content of the Sydney Phylogenetics Workshop has a generally linear structure, with each lecture and practical exercise building on the content of preceding workshop elements (Figure 2). Collectively, the lectures and practical exercises involve about 14 contact hours. The workshop begins with an introduction to tree thinking, including explanations and definitions of terms used in molecular evolution and phylogenetics. This introduction helps to ensure that all attendees are able to understand the foundational concepts and to use relevant and appropriate terminology when discussing phylogenetic trees and inference methods.

Participants are then introduced to the range of data types and fundamental evolutionary models that are used in phylogenetic analysis. After the principles of phylogenetic inference are described, the workshop introduces three of the major phylogenetic methods: maximum parsimony, distance‐based methods, and maximum likelihood. Although the basic frameworks of these methods are explained, their implementations are not described in depth; participants are referred to the literature should they wish to explore the theoretical and statistical underpinnings in detail (e.g., Felsenstein 2003; Yang 2014).

The second half of the workshop moves on to more advanced topics, including phylogenomics, Bayesian phylogenetic inference, and molecular dating. These are more conceptually challenging and build on the terminology, concepts, and principles described in earlier lectures and practical exercises. Given the time constraints, the treatment of advanced topics is necessarily limited, but participants are directed towards further online tutorials on more complex or specialized topics (e.g., Barido‐Sottani et al. 2018).

The content of the lectures is reinforced by elementary practical exercises that guide the participants through DNA sequence alignment, model selection, and phylogenetic analysis using distance‐based methods, maximum likelihood, and Bayesian inference. These practical exercises are designed to introduce widely used software for phylogenetic analysis, including MEGA (Kumar et al. 2024), IQ‐TREE (Bui et al. 2020), ASTRAL (Zhang et al. 2018), and BEAST (Bouckaert et al. 2019). The data analyses are based on biologically interesting case studies that can be reasonably completed within the scheduled class.

**FIGURE 3:**
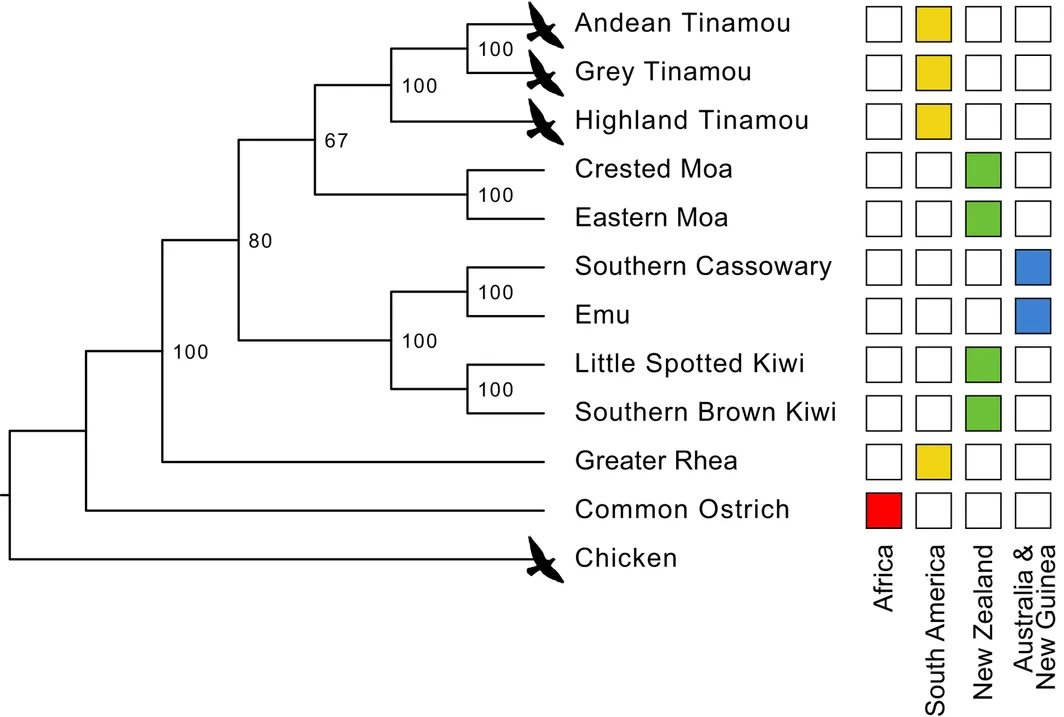
Phylogenetic tree of palaeognath birds inferred using DNA sequence data. The three species of tinamou are nested within the ratite group, with the chicken as an outgroup taxon. Silhouettes indicate species that have the ability to fly. Present‐day locations of the sampled ratite and tinamou species are indicated by the colored boxes on the right.

The first set of practical exercises focuses on the evolutionary history of ratites and tinamous, which together form the avian infraclass Palaeognathae. These birds provide a compelling case study because all living ratites are flightless, whereas their close relatives (tinamous) are able to fly. Under the assumption that ratites form a monophyletic group, the distribution of these traits would suggest a single loss of flight in the ancestral lineage leading to all living ratites (Cracraft 1974). Furthermore, the living ratites have a classic Gondwanan distribution throughout the land masses of the Southern Hemisphere, suggesting that they might have a deep history extending to the Mesozoic era when the supercontinent was still contiguous (Cracraft 1973; Haddrath and Baker 2001). Upon analyzing DNA sequence data from a selection of ratites and tinamous, the workshop participants find that the evolutionary history of palaeognaths is not necessarily consistent with the past views described above (Figure 3). The estimated evolutionary relationships are consistent with recent phylogenomic evidence (Stiller et al. 2024).

The remaining practical exercises are based on examples from Australasian marsupials and hominid evolution. The marsupial case study uses a subsample of a published phylogenomic data set (Duchêne et al. 2018), allowing workshop participants to investigate genuine questions about the evolutionary relationships among marsupial families. The hominid case study centers on the affinities of the ancient Denisovan humans, which are inferred using Bayesian phylogenetic analysis of mitochondrial genome sequences (Krause et al. 2010). Both of these examples are based on real data sets that have been scaled down to ensure computational feasibility, but that are still informative enough to produce well‐supported phylogenetic results that can be interpreted meaningfully. Upon completion of the practical exercises, workshop participants will have gained experience in a range of phylogenetic methods and models implemented in widely used software.

## 4 Lessons Learned and Improving Accessibility

The Sydney Phylogenetics Workshop has had a long‐term goal of improving accessibility to training opportunities, despite the lack of dedicated funding. The workshop is primarily intended for early career researchers in ecology and evolution, including those with a focus on molecular systematics, macroevolution, biogeography, and conservation biology. However, attendees have come from a variety of backgrounds and career stages, including undergraduate students about to commence their first research project. In recent years, large proportions of attendees have included biosecurity scientists, health scientists, pathologists, and molecular diagnosticians. The vast majority of workshop participants have a foundational level of knowledge, with 66% of attendees in 2025 having no or little experience in phylogenetics (with self‐reported scores of 1 or 2, based on a scale of 1 to 5). Only 3% of attendees considered their level of experience in phylogenetics to be high or advanced (scores of 4 or 5). Accordingly, the scope of the workshop has shifted to providing a broader introduction to phylogenetics.

We have taken a number of measures to improve the accessibility of the Sydney Phylogenetics Workshop, including the online provision of workshop materials and introducing the option of online attendance. Regardless of the financial circumstances or career stage of participants, attendance at the workshop is free for all registrants. However, budgetary limitations have prevented us from offering mobility grants to offset the costs of in‐person workshop attendance, such as travel and childcare expenses. Mobility grants would be instrumental in removing the most substantial barrier to in‐person attendance.

The option of online attendance has enabled much wider international participation in the workshop. Online attendees can watch the talks and post questions, though they are only able to receive limited assistance with the practical exercises. However, the practical exercises have detailed instructions and are designed to be largely self‐guided. Although the time zone of the workshop is suitable for attendees from the Asia‐Pacific region, it is unfavorable for those from other parts of the world, including Europe, Africa, the Middle East, and the Atlantic coast of the Americas. Furthermore, some attendees do not have steady access to good Internet connectivity, posing difficulties for watching live online lectures. These hindrances to online participation are not easily addressed, but lecture recordings are provided to registered participants via a private link to a shared folder. An additional possibility for improving accessibility would be to run repeat sessions that are suitable for different time zones, which is an option that we will consider in the future.

One of the guiding principles of the Sydney Phylogenetics Workshop is to provide a useful training and networking opportunity while being mindful of environmental impact. In this regard, all workshop content is freely available in electronic form, no merchandise is provided to participants, and materials are reused or recycled where possible. The organizers and instructors are based locally, minimizing the need for travel. The workshop's carbon footprint is also managed by prioritizing attendees from Australia and the Asia‐Pacific, while also providing an option for online attendance (Stroud and Feeley 2015; Jäckle 2022). International workshop participants are gently encouraged to attend online rather than in person. The workshop was held in a fully online format from 2020 to 2022 during the COVID‐19 pandemic, with a hybrid format formally introduced in 2025. All future workshops will be in a hybrid format, given that online attendance has a much smaller carbon footprint than in‐person attendance (Leochico et al. 2021; Spinellis and Louridas 2013; Tao et al. 2021).

From the perspective of event management, offering free workshop registration to all attendees comes with several disadvantages. Chief among these is the lack of an income stream for funding the workshop. However, the lack of a registration fee also means that a nontrivial proportion of applicants submit late cancellations or do not attend the workshop at all. This proportion has been ~10% among the applicants for in‐person attendance at the Sydney Phylogenetics Workshop, but higher for online attendees. A measure that considerably improved the rate of attendance was the introduction of an online application form, which allowed workshop organizers to offer places to selected attendees rather than simply admitting participants on a first‐come‐first‐served basis. Nevertheless, disparities between expected and realized attendance are an inevitable cost of holding a free event (Raby and Madden 2021a); we consider that this cost is considerably outweighed by the benefits and can be partially taken into account during planning.

## 5 Related Workshops

The Sydney Phylogenetics Workshop has extended its reach by providing teaching material for other courses and by forming the basis of related workshops. The workshop materials have been designed so that they can be delivered by anyone comfortable with the material, including early career researchers. A key benefit of having a complete set of curated workshop materials is that there is consistency in the usage of terminology, level of detail, and instructional examples, while also ensuring a logical progression of concepts and development of ideas.

There is no formal record of which courses have used the lecture slides and practical exercises, given that teaching material from the workshop is freely available online. However, some of the content has been used in specialized workshops on Bayesian phylogenetics, such as Taming the BEAST (Barido‐Sottani et al. 2018) and short courses on RevBayes (Höhna et al. 2016). Some courses and workshops have been held in close association with, or as derivatives of, the Sydney Phylogenetics Workshop. These include several workshops that have taken place at other Australian and international institutions. The workshop materials have been translated into Spanish and delivered in a course at Universidad del Rosario, Colombia. Teaching materials from the Sydney Phylogenetics Workshop are updated annually and we hope that they will continue to be used by researchers and instructors worldwide.

## 6 Concluding Remarks

Since its origins in 2010, the annual Sydney Phylogenetics Workshop has provided a training opportunity for more than 1000 students and researchers in the life, environmental, and health sciences. The syllabus and format of the workshop have been regularly revised in response to the needs and requests of participants, with various modifications having also been made to improve accessibility. Some of the key lessons learned from running the workshop have been the international scale of demand for introductory courses on phylogenetics, the feasibility of organizing and delivering an effective workshop even with minimal funding, and the substantial accessibility and sustainability benefits of the hybrid format. We expect that the workshop will continue to evolve in the coming years, particularly with expanding international participation enabled by its hybrid in‐person and online format. We hope that our workshop report provides some useful guidance for those who might consider organizing similar training events.

## Author Contributions

**Simon Y. W. Ho:** conceptualization (lead), funding acquisition (lead), project administration (lead), resources (lead), visualization (lead), writing – original draft (lead), writing – review and editing (lead).

## Conflicts of Interest

The author declares no conflicts of interest.

## Data Availability

Materials from the Sydney Phylogenetics workshop are available on Github: https://github.com/simon‐ho/SydneyPhyloWorkshop.
